# Effectiveness of Informed AI Use on Clinical Competence of General Practitioners and Internists: Pre-Post Intervention Study

**DOI:** 10.2196/75534

**Published:** 2026-02-05

**Authors:** Eyad A Qunaibi, Ayman M Al-Qaaneh, Baraa F Ismail, Hussam I Muhidat, Farhia S Rageh, Najwa A Musallam, Alaa K Fawzy

**Affiliations:** 1 Department of International Research MedOne Academy Dover, DE United States; 2 Faculty of Allied Medical Sciences Al-Balqa Applied University Al-Salt Jordan; 3 Faculty of Public Health Jinan University Tripoli Lebanon; 4 PriceWaterhouseCoopers Middle East Doha Qatar; 5 Fakeeh University Hospital Dubai United Arab Emirates; 6 King Hussein Cancer Center Amman Jordan; 7 Medical Biochemistry and Molecular Biology Department Faculty of Medicine Zagazig University Zagazig Egypt

**Keywords:** artificial intelligence, AI, clinical competence, artificial intelligence training, AI training, clinical decision support, medical education, digital health tools

## Abstract

**Background:**

Artificial intelligence (AI) shows promise in clinical diagnosis, treatment support, and health care efficiency. However, its adoption in real-world practice remains limited due to insufficient clinical validation and an unclear impact on practitioners’ competence. Addressing these gaps is essential for effective, confident, and ethical integration of AI into modern health care settings.

**Objective:**

This study aimed to evaluate the effectiveness of informed AI use, following a tailored AI training course, on the performance of general practitioners (GPs) and internists in test-based clinical competence assessments and their attitudes toward clinical AI applications.

**Methods:**

A pre-post intervention study was conducted with 326 physicians from 39 countries. Participants completed a baseline test of clinical decision-making skills, covering diagnosis, treatment planning, and patient counseling; attended a 1.5-hour online training on effective AI use; and then took a similar postcourse test with AI assistance permitted (GPT-4.0). Test performance and time per question were compared before and after the training. Participants also rated AI accuracy, efficiency, perceived need for structured AI training, and their willingness to use AI in clinical practice before and after the course.

**Results:**

The average test scores improved from 56.9% (SD 15.7%) to 77.6% (SD 12.7%; *P*<.001), and the pass rate increased from 6.4% (21/326) to 58.6% (191/326), with larger gains observed among GPs and younger physicians. All skill domains (diagnosis, treatment planning, and patient counseling) improved significantly (all *P*<.001), while time taken to complete the test increased slightly from before to after the course (mean 40.25, SD 16.14 min vs 42.29, SD 14.02 min; *P*=.03). By the end of the intervention, physicians viewed AI more favorably, reporting increased confidence in its accuracy and time efficiency, greater appreciation for the need for structured AI training, and increased confidence and willingness to integrate AI into patient care.

**Conclusions:**

Informed use of AI, based on tailored training, was associated with higher performance in test-based clinical decision-making assessments and greater confidence in using AI among GPs and internists. Building on previous research that often lacked structured training, focused primarily on model performance, or was limited in clinical scope, this study provides empirical evidence of both competence and perceptual improvement following informed AI use in a large, multinational cohort, enhancing the generalizability. These findings support the integration of structured AI training into medical education and continuing professional development to improve clinical performance and promote competent use of AI in clinical practice.

## Introduction

Artificial intelligence (AI) has demonstrated significant potential in disease diagnosis, treatment recommendations, patient engagement, and medical writing and education [1-3]. Extensive research has explored and compared the accuracy of AI models across these domains [4-7]. In addition, AI has revolutionized diagnostic radiology by enhancing image analysis and interpretation [8]. However, despite these promising applications, most AI models and software remain within the realm of research rather than real-world settings, reflecting a notable gap in implementation [9,10].

Before widespread adoption, AI applications in health care require not only algorithm validation but also clinical validation, comparing AI-based interventions with standard treatments and existing clinical practices [11]. A limited number of studies have evaluated the effect of AI assistance in clinical settings and demonstrated improvements in the quality, efficiency, and effectiveness of health care services [12,13]. In contrast, there is an abundance of studies that have evaluated the impact of AI assistance in controlled experimental settings rather than real-world clinical scenarios. Feigerlova et al [14] highlighted the limitations of these studies, including small sample sizes, single-center designs, inadequate control for confounding factors, and the absence of well-defined clinical competence frameworks.

In addition, existing studies often do not include training on appropriate AI use. To enable AI integration into clinical practice, tailored AI training for health care practitioners is required [15]. The situation is even more critical for primary care physicians, as Liaw et al [16] have highlighted the lack of attention given to training primary care physicians in the use of AI-based tools and emphasized the necessity of such training to maximize benefits and minimize potential harms. Ideally, this training should be followed by objective assessments of improvements in clinical competence after AI use. However, evidence on the objective improvements in clinical competence with AI assistance after structured AI training remains scarce.

Beyond technical accuracy and the need for training, the successful adoption of AI in clinical settings is heavily influenced by health care professionals’ perceptions and attitudes. Acceptance is an indispensable prerequisite for the widespread implementation of AI [15]. Although many studies have assessed health care professionals’ general attitudes toward AI integration into medical practice [15], to the best of our knowledge, no study has specifically examined how tailored AI training impacts practitioners’ perceptions of AI and their willingness to incorporate it into clinical workflows. A recent systematic review identified key factors that influence health care workers’ trust in AI tools for making informed clinical decisions [17]. However, none of the 27 included studies implemented interventions with pre-post repeated-measurement designs; instead, their assessment methods were predominantly semistructured qualitative interviews, focus groups, or Likert-scale cross-sectional surveys. Collectively, many previous studies were limited by small sample sizes, narrow clinical scope, a lack of structured AI training, evaluation in simulated rather than real-world clinical contexts, and a lack of pre-post intervention assessments of perceptual change. These accumulated limitations and gaps in knowledge highlight the significance of our study.

Unlike previous research, our study approximates real-world clinical practice by integrating AI as a decision-support tool for general practitioners (GPs) and internists who were tested using real clinical case scenarios. Through a structured, tailored training course with systematic pre-post course assessments, we objectively evaluate test-based clinical competence of GPs and internists following informed AI use. We assess improvements in the skills of diagnosis, management, and patient counseling. We also uniquely assess shifts in GPs’ and internists’ perceptions of AI diagnostic accuracy, treatment recommendation efficacy, time efficiency, confidence, and willingness to integrate AI into clinical practice. In addition to these novelties, our study is multinational, large-scale, and outcome-driven, addressing key limitations of previous research.

By providing a replicable model for evaluating AI-assisted clinical decision-making, this study establishes a framework for optimizing AI use in health care settings, helping to bridge the gap between research-based AI performance and real-world clinical effectiveness. The aim of this study was to evaluate the test-based clinical competence and perceptions of GPs and internists, following informed AI use after a tailored AI training course.

## Methods

### Study Design and Procedures

This quasi-experimental study used a pre-post test design to evaluate the impact of informed AI use on clinical competence among GPs and internists. Conducted entirely online, the course and assessments were asynchronous, allowing participants to complete the training and tests at their preferred time. Each participant completed a precourse test before accessing the recorded modules, followed by a postcourse test, in which AI assistance was permitted (ChatGPT [version 4.0; OpenAI]), to measure changes in clinical competence. In this study, *informed AI* *use* refers to physicians using AI tools after receiving a tailored AI training course on how to effectively and responsibly integrate these tools into their practice. Participants’ self-reported previous use of AI in clinical practice was recorded and categorized for analysis (Multimedia Appendix 1).

### Ethical Considerations

This study was conducted in accordance with ethical guidelines and approved by the institutional review board of MedOne Academy (MO-IA-24/25-EDU-1). All participants were informed about the study objectives and the voluntary nature of their participation, with the right to withdraw at any time. Electronic informed consent was obtained, and participants were assured of data confidentiality. Participants’ names and email addresses were collected solely for administrative purposes to assign and link precourse and postcourse assessments. These identifiers were not used for analysis and were not shared with any third party. All study data were analyzed in coded and anonymized form. Access to identifiable information was strictly limited to the first and corresponding authors. All data were stored on secure, password-protected systems accessible only to authorized members of the research team. Participants did not receive any financial or material compensation for participation in this study.

### Participants

Participants included GPs, encompassing family medicine specialists and internists, including those pursuing subspecialty training after internal medicine. These classifications apply throughout the study. Details of participant recruitment, eligibility verification, and enrollment timeline are provided in Multimedia Appendix 2.

### Assessment Tool

#### Overview

This study used a structured assessment tool comprising multiple-choice questions (MCQs) designed to evaluate clinical competence across three key areas: (1) diagnosis and patient assessment, (2) treatment planning and personalized medicine, and (3) patient counseling. Each test set (A and B) included items covering these same domains to ensure content consistency across assessments. The MCQs were developed by 2 of the study authors (AKF and BFI), who are physicians, and reviewed and revised by 2 additional expert physicians (Kamel Hatahet and Suleiman Al Ashi), to ensure their relevance to participants and the adequacy in assessing the 3 studied competencies. The test sets underwent expert review for face and content validity, and their clarity and timing were further verified in the pilot phase as described subsequently. Multimedia Appendix 3 includes the source references from which the clinical cases were derived, along with selected adapted sample cases and their corresponding MCQs with answer keys.

#### Crossover Design and Validation

A crossover design was implemented, with participants allocated to 2 groups for the precourse test: group A received 25 MCQs, and group B received 23 MCQs of comparable difficulty. Participants who registered through the MedOne Academy platform were alternately allocated to group A or group B to maintain balanced group sizes and comparable baseline characteristics. No significant difference was observed in the GP-to-internist distribution between the 2 test-order groups (*P*=.11), supporting the comparability of participant composition. After the course, participants switched question sets. This crossover design was selected because it allowed each participant to serve as their own control, thereby reducing variability due to individual differences and maintaining statistical power with a feasible sample size while ensuring that all participants received the educational intervention. Conducting a conventional randomized controlled design would have required a nonintervention control group, which was impractical and would likely have compromised motivation and completion rates, as the course itself served as the primary educational and engagement incentive.

The difficulty level was preassessed by the 4 contributing experts (AKF, BFI, Kamel Hatahet, and Suleiman Al Ashi) to ensure equivalence, and the comparability of participant performance across both test sets A and B was statistically confirmed, as detailed in the Results section and Table S1 in Multimedia Appendix 4.

#### Assessment Administration and Scoring Criteria

**Figure 1:**
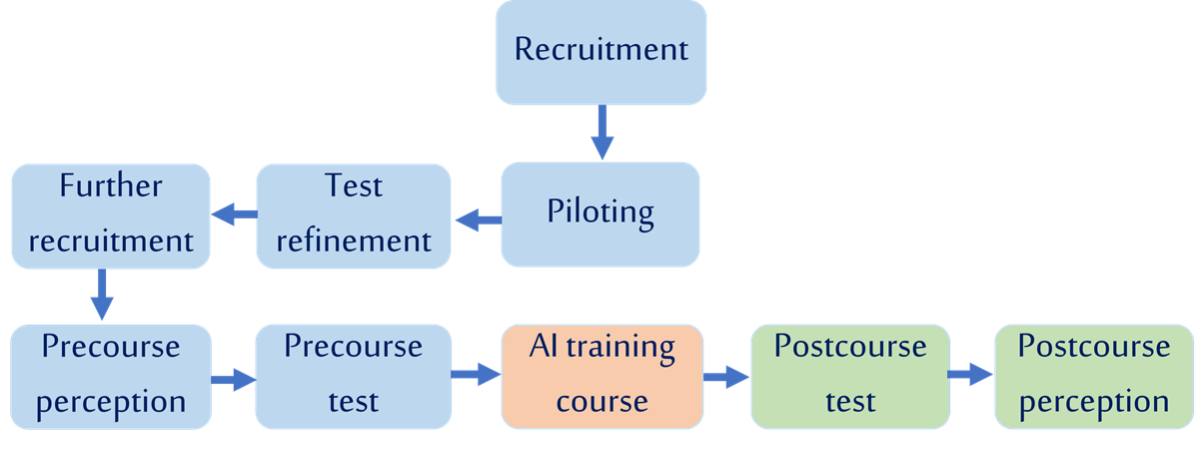
Study flow diagram. Participant recruitment and piloting (including test refinement), followed by precourse perception survey and competence test, artificial intelligence training course, and postcourse competence test and perception survey. Blue indicates preparatory phases, orange the intervention, and green postintervention assessments.

Each test was allotted a maximum duration of 1 hour. A passing threshold of 80% was applied, consistent with established benchmarks in medical certification (Multimedia Appendix 5). Clear and standardized instructions on AI use were provided to participants to ensure uniform conditions across assessments. These instructions, along with the technical setup implemented to maintain the reliability of experimental conditions, are detailed in Multimedia Appendix 6. Additionally, participants completed the same set of perception questions before and after the course to assess how the course and study experience influenced their attitudes toward AI in clinical practice. These questions assessed participants’ perceptions of AI accuracy in diagnosis and treatment planning, its time-saving potential in clinical settings, the perceived need for structured AI training, and their willingness and confidence to incorporate AI into clinical practice (Multimedia Appendix 7). Each participant was given up to 2 weeks to complete the entire study process after enrollment. A summary of the study flow is illustrated in Figure 1.

GPT-4.0 was selected as the designated large language model (LLM) for participants to use in the postcourse test. Its selection was based on published evidence demonstrating the superior performance of GPT models on medical examination–style questions compared with other contemporary LLMs [7].

### Intervention (Tailored AI Course)

The training program, titled AI Skills in Medicine, was designed as an asynchronous 1.5-hour online workshop prepared and presented by the main author (EAQ). It introduced AI applications in diagnosis, management, predictive analytics, and triage and emphasized ethical and responsible implementation. The course also included brief segments on privacy, account customization, and optimal AI use in clinical reasoning. Participants were instructed to evaluate AI outputs and their underlying rationale carefully rather than accepting them as is. This approach aligns with the viewpoint of Izquierdo-Condoy et al [18], who argued that, when thoughtfully embedded within educational frameworks, generative AI tools can enhance cognitive abilities, supporting rather than replacing clinical reasoning. By addressing these various modules, the program covered the competencies suggested by previous research [16] to maximize potential benefits and minimize harms associated with AI incorporation in clinical practice. A detailed outline of the 4 modules and learning objectives is provided in Multimedia Appendix 8.

### Pilot Study

Before the main study, a pilot group of 15 GPs and internists was recruited to follow the same study procedure, including attending the course and completing the tests. This phase allowed researchers to evaluate the clarity and timing of the tests as well as the applicability of the test platform. On the basis of participant feedback, the assessment process and training content were refined. For instance, open-ended questions were removed after observing excessive time consumption and noting that poor scores often reflected time constraints rather than knowledge gaps. The 15 pilot participants were excluded from the final analysis.

### Studied Parameters

#### Primary Outcome: Clinical Competence Assessment

This study assessed multiple parameters to evaluate the impact of informed AI use comprehensively. Primary outcomes were the clinical competence scores obtained in the precourse and postcourse tests, the difference between postcourse and precourse scores, the pass or fail status based on an 80% threshold in both tests, and the total test completion time (min) before and after the course.

#### Secondary Outcomes: Skill-Specific Performance Measures

Secondary outcomes focused on skill-specific performance and included the number of correct answers per skill domain (diagnosis, treatment planning, and patient counseling) and the average time per correct answer per skill. The latter was calculated by summing the time spent on correct answers across all relevant case scenarios within each skill domain and dividing by the number of correct responses.

#### Perception Outcomes and Assessment

Perceptual changes were also evaluated, covering participants’ perceptions of AI accuracy in diagnosis and treatment planning, its time-saving potential, the perceived benefit of structured AI training, and their willingness to incorporate AI into clinical practice. The perception shift was defined as the change in responses to these perception items before and after the intervention.

All outcomes were analyzed to determine improvements in clinical competence and shifts in attitudes following the tailored AI training and informed AI use.

### Statistical Analysis

Categorical variables were presented as frequencies and percentages, and continuous variables were reported as means with SDs or medians with IQRs, depending on data distribution assessed using the Kolmogorov-Smirnov test. Baseline differences in categorical variables were analyzed using the chi-square goodness-of-fit test, whereas the Wilcoxon signed-rank test (Mann-Whitney U test) or the independent samples *t* test was applied for continuous variables, as appropriate. The validity of the crossover design was assessed using the chi-square test, Mann-Whitney *U* test, or independent sample *t* test, as appropriate. The McNemar test or paired-samples *t* test was used to evaluate differences in the number of participants who passed or failed, test scores (%), and completion time (min) before and after AI use, as appropriate. An independent samples *t* test or 1-way ANOVA determined which group benefited most from AI assistance. Spearman rank correlation assessed the association between participants’ age and score differences. The Wilcoxon signed-rank test with effect size analysis was used to evaluate changes in scores, time, and perceptions before and after AI use, stratified by skill domains (diagnosis, treatment planning, and patient counseling). Perception differences between groups were analyzed using the Mann-Whitney *U* or Kruskal-Wallis test, as appropriate. All statistical tests were 2 tailed, and statistical significance was set at *P*<.05. No imputations were made for missing data points. All data used in the study were analyzed using SPSS (version 25.0; IBM Corp).

## Results

### Recruitment and Baseline Characteristics

**Figure 2:**
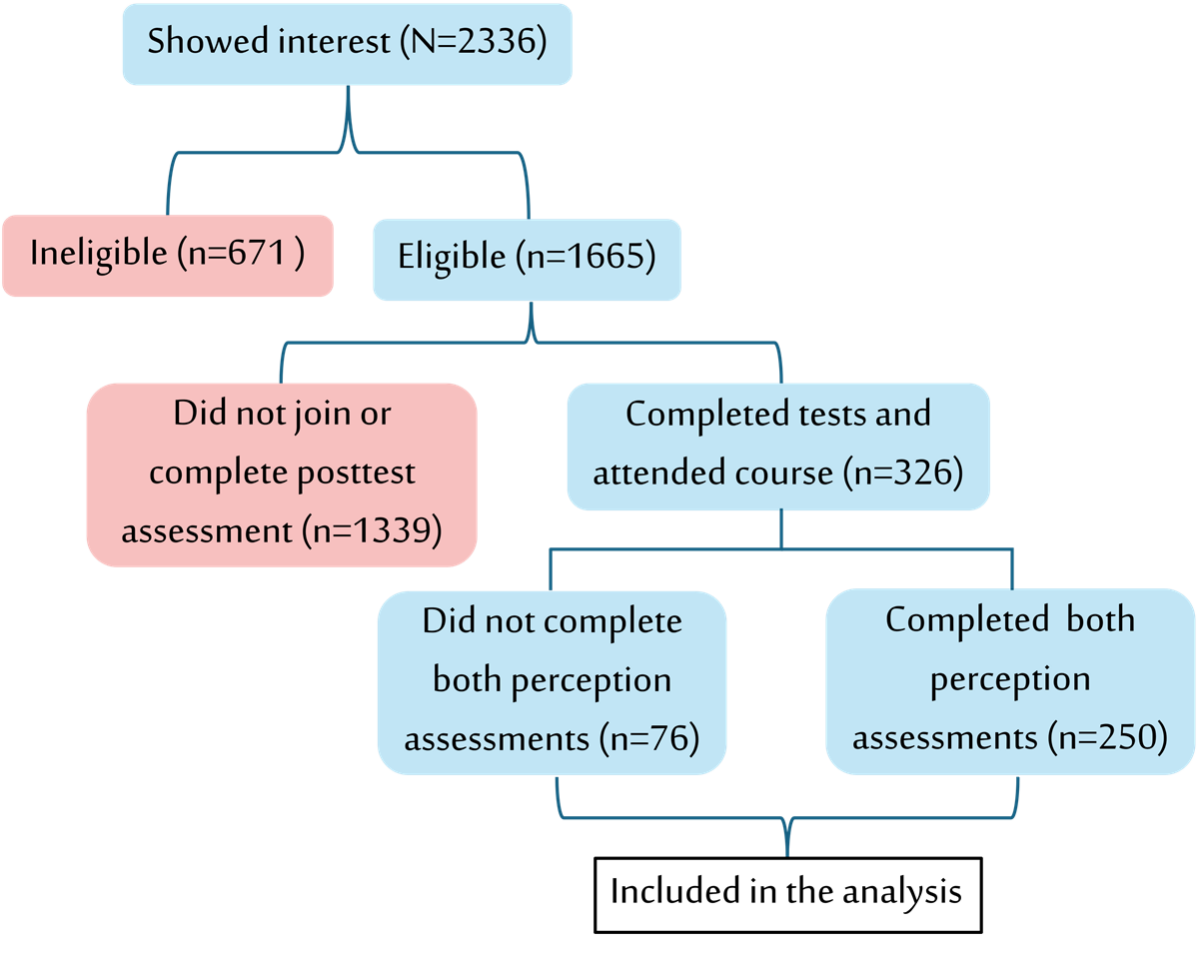
Recruitment, eligibility screening, and participant inclusion flowchart. Of 2336 individuals who expressed interest, 671 (28.7%) were ineligible and 1665 (71.3%) were eligible. Among eligible individuals, 1339 (80.4%) did not enroll or did not complete the postcourse test, and 326 (19.6%) completed both tests and attended the artificial intelligence training course. Of these, 76 (23.3%) did not complete both perception assessments and 250 (76.7%) completed all study components. Red boxes indicate attrition or exclusion at each stage.

A total of 2336 individuals completed the recruitment form, of whom 1665 (71.3%) met the eligibility criteria by belonging to the target specialties and providing valid medical licensure. The remaining 671 (28.7%) individuals were excluded for lacking licensure or for being medical students, specialists, or professionals outside the core medical field. Of the eligible 1665 participants, 326 (19.6%) completed the precourse test, attended the course, and took the postcourse test. Of the 326 participants, 250 (76.7%) also completed the precourse and postcourse perception assessments. All 326 participants were included in the final analysis (Figure 2).

Table 1 summarizes the demographics of the 326 participants (male: n=193, 59.2%; median age 31, IQR 27.75-38 y). Most (n=229, 70.2%) were GPs. Most participants (n=208, 63.8%) reported little to no previous use of AI in clinical practice, and 308 (94.5%) had no previous AI training. Nearly half (n=173, 53.1%) served both inpatients and outpatients. The baseline median score on the precourse test was 57% (47.9%-69.6%), with a median completion time of 41.0 (27.0-56.0) minutes. The participants were distributed across 39 countries, with the highest participation from Saudi Arabia, Syria, Egypt, Algeria, and Jordan.

**Table 1:** Participant demographics and baseline characteristics (n=326)^a^.

Variable		Values
**Sex, n (%)**		
	Male	193 (59.2)
	Female	133 (40.8)
**Specialty, n (%)**		
	GP^b^	229 (70.2)
	Internist	97 (29.8)
**Use of AI^c^ in clinical practice, n (%)**		
	Not at all	139 (42.6)
	Rarely	69 (21.2)
	Sometimes	87 (26.7)
	Often	18 (5.5)
	Almost every day	13 (4)
**Familiarity with AI, n (%)**		
	GP (n=229)	84 (36.7)
	Internist (n=97)	34 (35.1)
**Received previous training in AI use, n (%)**		
	Yes	18 (5.5)
	No	308 (94.5)
**Type of patients served, n (%)**		
	Outpatients	96 (29.4)
	Inpatients	57 (17.5)
	Both	173 (53.1)
**Test results before the AI** **training** **course, n (%)**		
	Fail	305 (93.6)
	Pass	21 (6.4)
**Age (y), median (IQR)**		31.00 (27.75-38.00)
	GPs	30.00 (27.00-37.00)
	Internists	35.00 (29.00-40.00)
**Precourse score (%), median (IQR)**		57.00 (47.85-69.60)
	Precourse score of GPs	56.00 (44.00-64.00)
	Precourse score of internists	68.00 (52.00-74.00)
Time taken for precourse test (min), median (IQR)		41.00 (27.00-56.00)

^b^GP: general practitioner.

^c^AI: artificial intelligence.

***^a^***Data are presented as counts (percentages) for categorical variables and as medians (IQRs) for continuous variables.

### Validation of the Crossover Design

To validate the crossover design, precourse scores (*P*=.65), postcourse scores (*P*=.65), and score differences (*P*=.15) were compared between groups A and B, along with pass and fail comparisons between test sets A and B at both time points (pretest: *P*=.31; posttest: *P*=.19; Table S1 in Multimedia Appendix 4). None of these comparisons yielded a statistically significant difference, confirming the validity of the study design.

### Effect of the Informed AI Use on Participants’ Competence

Table 2 illustrates the impact of informed AI use on participants’ competence. The proportion of participants who passed the test after taking the course and being allowed to use AI increased significantly (*P*<.001). The mean score improved from 56.88% (SD 15.65%) to 77.56% (SD 12.71%), with a mean difference of 20.68% (*P*<.001). The mean time taken to complete the test increased slightly (by approximately 2 minutes) from before to after the course (40.25, SD 16.14 vs 42.29, SD 14.02 minutes; *P*=.03).

**Table 2:** Effect of informed artificial intelligence use on the participants’ competencies (n=326). Comparison of participants’ performance before and after the artificial intelligence training course.

Variable			Before course		After course		Mean difference		Test statistic			*P* value	
**Test results, n (%)**										*χ*^2^_1_=153.55^a^			<.001
	Fail	305 (93.6)		135 (41.4)		—^b^							
	Pass	21 (6.4)		191 (58.6)		—							
Score (%), mean (SD)			56.88 (15.65)		77.56 (12.71)		20.69		*t*=_325_–19.58^c^			<.001	
Time taken (min), mean (SD)			40.25 (16.14)		42.29 (14.02)		2.04		*t*=_325_–2.17^c^			.03	

^a^McNemar *χ*^2^.

^b^Not applicable.

^c^Paired samples *t* test.

Score improvements were analyzed across participant characteristics (Table S2 in Multimedia Appendix 4), revealing no significant differences except between GPs and internists, with the former showing significantly greater improvement than the latter (23.7%, SD 19.1% vs 13.7%, SD 17.3%; *P*<.001). Spearman correlation analysis revealed a statistically significant weak negative correlation between participants’ age and score difference (ρ=–0.143; *P*=.01).

### Skill-Specific Performance Outcomes

**Figure 3:**
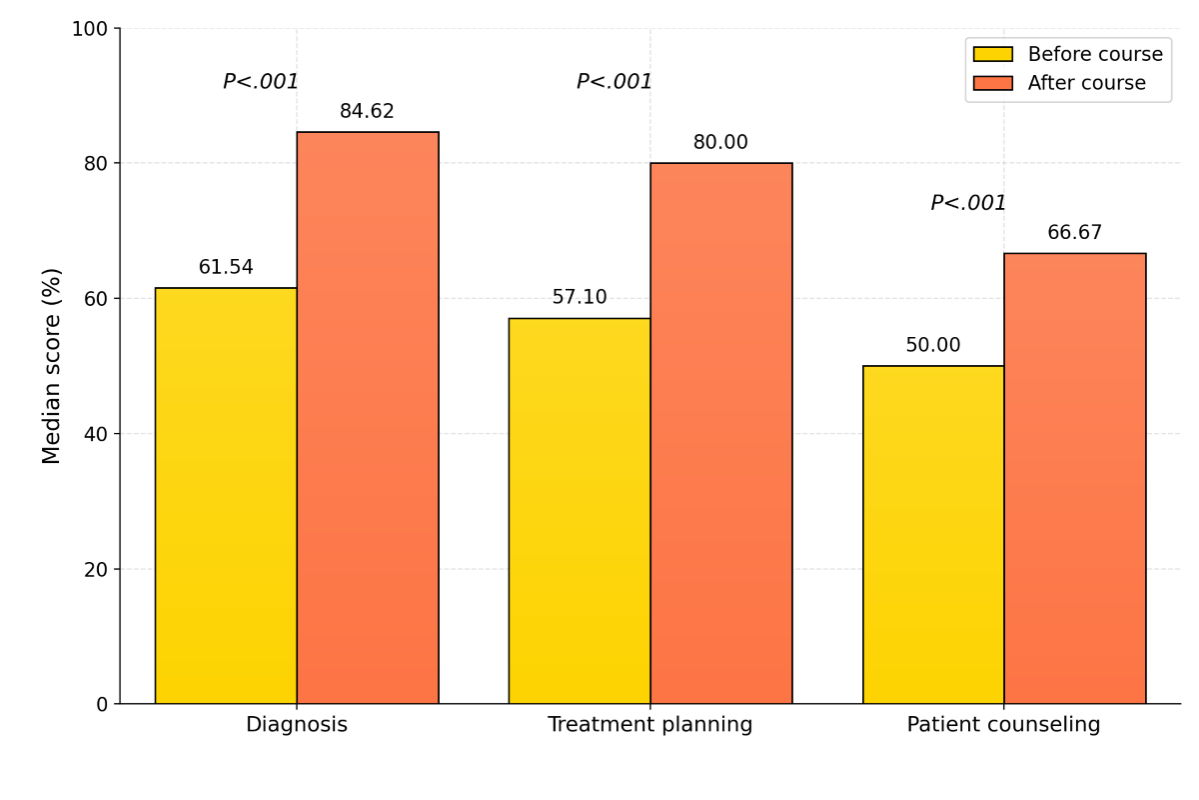
Skill-specific median scores before and after informed artificial intelligence (AI) use (N=326). Median scores (% for diagnosis, treatment planning, and patient counseling before and after the AI training course. Wilcoxon signed-rank tests showed statistically significant differences for all skills (*P*<.001).

To determine whether the observed improvement was broad across all competencies or specific to certain skill areas, individual skill-specific performance was analyzed. Significant score increases were observed across skills of diagnosis, treatment planning, and patient counseling (Table 3; Figure 3; *P*<.001 for all). However, the average time per correct answer per skill increased significantly after informed AI use (Table S3 in Multimedia Appendix 4).

**Table 3:** Score comparison for each skill (n=326)^a^.

Skill	Precourse score (%), median (IQR)	Postcourse score (%), median (IQR)	*z* score	*P* value^b^	Effect size, *r*
Skill 1: diagnosis	61.54 (46.15-69.23)	84.62 (76.90-92.30)	–12.57	<.001	0.738
Skill 2: treatment planning	57.10 (42.14-70.00)	80.00 (70.00-85.71)	–12.39	<.001	0.686
Skill 3: patient counseling	50.00 (33.33-100.00)	66.67 (50.00-100.00)	–7.75	<.001	0.420

^a^Comparison of participants’ precourse and postcourse scores across 3 assessed competencies: diagnosis, treatment planning, and patient counseling. Data are presented as median (IQR).

^b^Wilcoxon signed-rank test.

### Effect of the AI Course and Informed AI Use on Participants’ Perceptions

**Figure 4:**
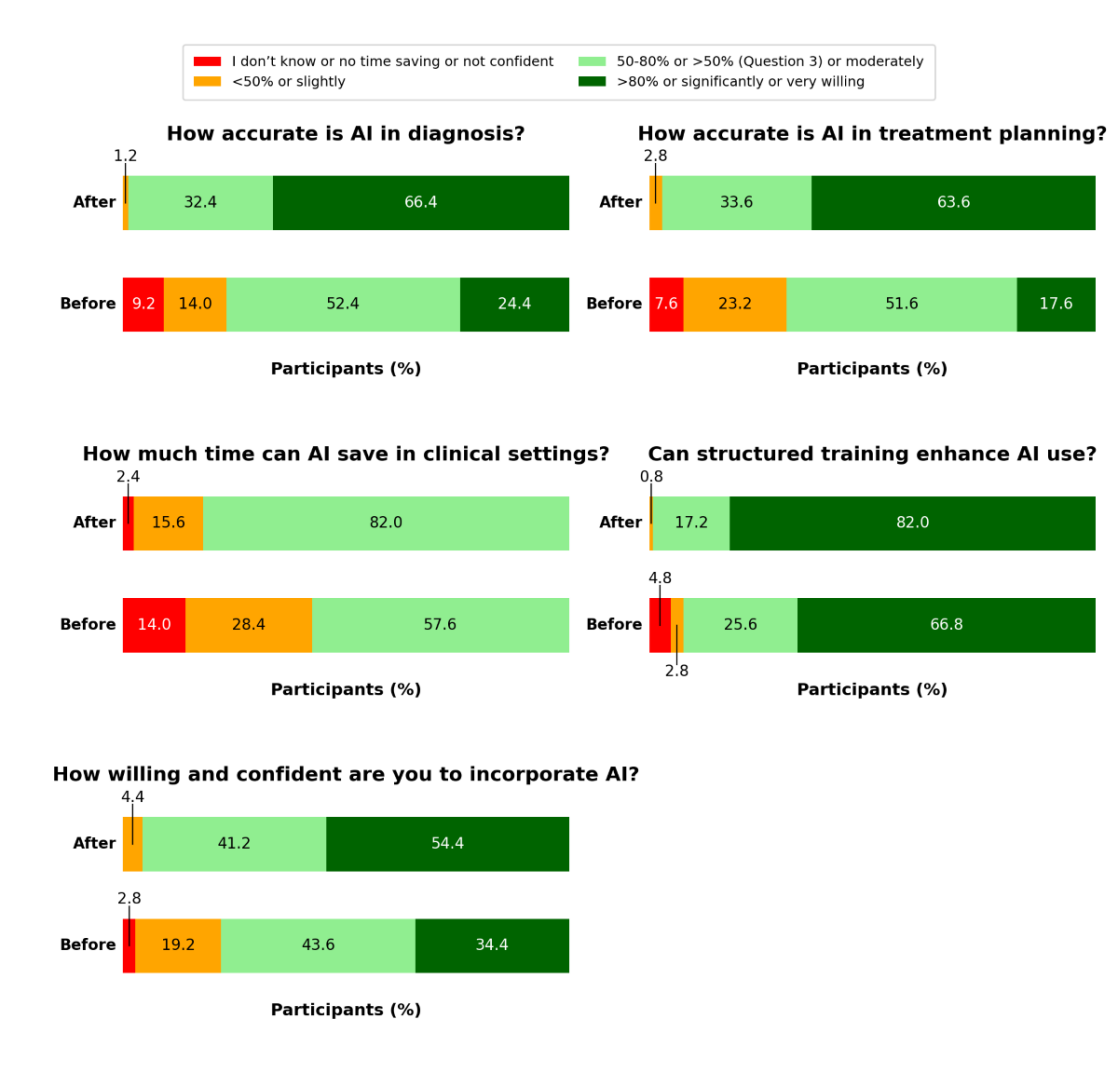
Changes in participants’ perceptions of artificial intelligence (AI) before and after the course. Stacked bar charts show participants’ responses to 5 perception questions administered before and after the AI training course. Each bar represents the percentage of participants selecting each response category. Dark green indicates very positive perceptions (>80%, significantly, or very willing), light green indicates intermediately positive perceptions (50%-80% or moderately; >50% for question 3), orange indicates conservative perceptions (<50% or slightly), and red represents uncertain or negative responses (“I do not know,” “no time saving,” or “not confident”).

As shown in Figure 4 and Table S4 in Multimedia Appendix 4, participants’ perceptions of AI accuracy, time efficiency, and integration into clinical practice improved markedly in the postcourse perception assessment, reflecting changes observed after both the course and informed AI use. All 5 perception changes (questions 1-5) were statistically significant (Table S5 in Multimedia Appendix 4). The proportion of participants who believed AI to be highly accurate in diagnosis (question 1) and treatment planning (question 2) (>80% of the time) increased substantially. More participants also recognized AI’s time-saving potential (question 3), with a significant shift toward believing that AI can reduce health care professionals’ workload by 50% or more. Additionally, structured AI training (question 4) was perceived as highly beneficial, with a notable rise in participants rating it as significantly effective. Confidence in and willingness to incorporate AI into clinical practice (question 5) also increased, with a greater proportion of participants feeling very willing and confident after the course and informed AI use.

No significant difference in perception shift was observed based on participants’ sex, specialty, type of patients served, or previous AI training. However, participants who were unfamiliar with AI before the course exhibited a greater shift in their perception of AI diagnostic accuracy (*P*=.01) and greater willingness and confidence to incorporate AI tools into clinical practice (*P*<.001; Table S6 in Multimedia Appendix 4).

## Discussion

### Principal Findings

This study aimed to examine the effect of informed AI use, following a tailored course, on test-based clinical decision-making and the perceptions of GPs and internists. We have shown that informed AI use was associated with higher clinical decision-making scores using clinical scenarios that simulated daily practice. Furthermore, participants reported higher perceived AI accuracy as a diagnostic and therapeutic assistant, increased perceived time-saving efficiency, a stronger belief in the need for structured AI training programs, and greater confidence in incorporating AI into clinical practice.

Our study has several strengths and novel elements. It included structured, tailored AI training; assessed both objective changes in competence and subjective shifts in perceptions; and specifically targeted GPs and internists, a group previously underrepresented in AI research [19]. Additionally, it uniquely bridged education and objective assessments through structured precourse and postcourse tests. Moreover, this study was multinational and large scale, and it covered a comprehensive range of clinical competencies rather than a single isolated skill, addressing key limitations cited in previous research [14]. However, because participation was voluntary and completion required finishing both assessments, the analyzed sample likely represents a more AI-motivated subset of eligible registrants, which may limit the generalizability of the observed effects.

Furthermore, this study directly addresses a widely recognized training gap in medical education. Oftring et al [20] emphasized that the growing number and impact of medical AI applications necessitate more AI-focused curricula and research on their educational impact, as most current practitioners and trainees remain underprepared for AI integration into clinical practice. Similarly, Ichikawa et al [21] found that US Colleges of Osteopathic Medicine largely lack AI policy guidance or training for students and faculty, highlighting the urgent need to implement need-driven training in their programs.

The observed improvement in competence scores (57%-78%) is substantial. Whether AI assistance alone, without structured training, can reliably enhance physicians’ performance remains debated. AI has been shown to improve diagnostic accuracy in specific tasks such as detecting breast lesions on ultrasound [22], increasing fracture detection sensitivity [23], and interpreting pediatric radiographs [24]. However, Goh et al [25], in a study closest to ours in terms of participant specialties, found that unguided access to LLMs did not improve overall diagnostic reasoning among family medicine, internal medicine, or emergency medicine physicians.

Overall, the substantial improvement observed in our study is unlikely to be attributable to AI use alone, separately from the tailored course. Our training course focused on informed use of AI as a decision-support tool, helping participants double-check their reasoning, consider broader differentials, interact with AI-generated answers, and retrieve relevant literature and clinical guidelines, ultimately enhancing their responses to clinical scenarios. In addition, the substantial improvement in scores cannot be attributed to increased test-taking time, as completion time increased only slightly after the course, indicating that GPs and internists became more accurate with minimal impact on efficiency.

In contrast, a large-scale study by Yu et al [26] involving 140 radiologists and including training on AI use reported heterogeneous effects of AI assistance across 15 chest X-ray diagnostic tasks. Although the study specified that physicians received onboarding training on the AI system, it did not provide details on its content or structure. It is possible that a more clearly structured and tailored training approach, such as the one implemented in our study, might have produced more consistent improvements.

An interesting finding was that GPs demonstrated significantly greater improvement in test scores than internists (23.7% vs 13.7%, respectively). Given that baseline familiarity with AI was comparable between both groups, this discrepancy may be partly explained by the significantly lower baseline scores among GPs, which allowed a wider margin for improvement. GPs were significantly younger than internists, and younger generations are digital learners, engage more frequently in online communities [27], and use technology more fluently than older cohorts [28]. This interpretation also aligns with our findings of greater score improvement among younger participants and reflects the relatively young age of our study cohort (median 31.00 y).

The observation of no significant difference in participant score improvement based on familiarity with AI use in clinical practice aligns with findings reported by Yu et al [26] among radiologists, in which familiarity with AI tools failed to reliably predict the impact of AI assistance.

One surprising finding was the increased time per correct answer across all 3 competencies (diagnosis, treatment planning, and patient counseling) after AI use. This may reflect more deliberate and reflective reasoning, as participants were instructed to verify AI-generated suggestions and references against their own clinical judgment rather than accept outputs uncritically. Additionally, many participants were new to structured AI use; most (308/326, 94.5%) had no previous AI training, and approximately two-thirds (208/326, 63.8%) reported little to no previous AI use. Therefore, a short adaptation period was anticipated during their first guided application of AI in case-based testing.

The crossover design minimized familiarity bias and was validated by demonstrating no significant difference in precourse scores, postcourse scores, score improvements, and proportions of pass versus fail between the 2 test-order groups. This approach enhanced internal validity by enabling each participant to serve as their own control.

Beyond objective competence improvements, the observed changes in participants’ perceptions of AI’s accuracy, time efficiency, and potential integration into clinical practice emphasize the importance of structured training and guided AI use in realistic clinical scenarios. Following the intervention, the percentage of participants who perceived high accuracy (>80%) of optimally used AI platforms in diagnosis and treatment planning domains nearly tripled. This finding aligns with Abbas et al [7], who reported comparable accuracy of GPT models on National Board of Medical Examiners clinical subject examination questions.

The notable postcourse increase in physicians’ willingness and confidence to incorporate AI into clinical practice reflects one of the most impactful outcomes of this study, suggesting the potential value of structured AI training. The systematic review by Tun et al [17] identified training and familiarity as perceived facilitators of clinician trust in AI tools in making informed clinical decisions. Our data empirically demonstrate this effect. If health professionals lack sufficient trust in AI tools, they may disregard their recommendations, limiting the potential to enhance patient outcomes and optimize clinical workflows [21]. Through its tailored AI course, this study aimed to help participants understand AI capabilities and limitations, enabling them to integrate its assistance beneficially neither erroneously ignoring its outputs nor following them uncritically.

A novel aspect of our study is its pre-post assessment of attitude changes following the intervention, which, to our knowledge, has not been previously applied among physicians in the context of AI integration. Our study uniquely provides evidence linking structured training and competence improvement with enhanced perception of AI, highlighting the potential of targeted educational interventions to promote informed AI acceptance in clinical practice.

### Implications and Future Directions

Our study highlights the potential of informed AI use after a tailored AI training course to enhance GPs’ and internists’ diagnostic and management skills. Despite the Federation of State Medical Boards advocating for AI competence in medical education, structured AI training remains largely absent [19]. Similarly, 82% of medical professionals in a survey by Tezpal et al [29] recognized the need for AI education. To address this gap, AI training should be integrated into medical education, residency programs, and continuing medical education to ensure responsible and effective AI use. Given the rapid evolution of AI technologies, ongoing updates and refresher courses are essential.

Future research should investigate whether these test-based competence gains are sustained over time and whether they translate into measurable improvements in patient outcomes and clinical decision quality. Finally, further research should explore customized AI training for specialties such as emergency and critical care medicine, given the crucial role of timely and accurate decisions in these specialties.

### Limitations

Our study design does not allow isolation of the effect of the tailored AI training course from AI use without training, as it did not include a nonintervention control arm. Adding a third arm comparing informed versus noninformed AI use was impractical due to expected low motivation and completion rates among participants asked to repeat time-intensive tests without the educational incentive. A similar approach was taken in previous research assessing AI assistance among 140 radiologists, in which onboarding training was not isolated from AI impact [26].

Our participants were already inclined toward AI adoption, as evidenced by their willingness to enroll in our time-intensive study. This suggests that their baseline openness to AI was likely higher than that of physicians more broadly. Thus, the observed shift may underestimate the potential impact of informed AI use based on a tailored AI training course.

### Conclusions

This study provides evidence that informed AI use, guided by a tailored AI course, is associated with higher clinical decision-making competence among GPs and internists in test-based clinical scenarios. Beyond higher scores across diagnosis, treatment planning, and patient counseling domains, participants reported higher perceived AI diagnostic and therapeutic accuracy, greater perceived time efficiency, and increased willingness and confidence to integrate AI as a decision-support tool into clinical practice. These findings support the integration of structured AI training into medical education and continuing professional development to facilitate informed and responsible AI use, with plausible potential to improve patient care.

## Appendix

Multimedia Appendix 1 Classification of previous artificial intelligence use.

Multimedia Appendix 2 Recruitment of participants.

Multimedia Appendix 3 Clinical case scenarios, assessment questions, and source references.

Multimedia Appendix 4 Additional statistical tables and supporting results.

Multimedia Appendix 5 Passing score rationale.

Multimedia Appendix 6 Artificial intelligence use instructions and technical setup.

Multimedia Appendix 7 Evaluation of participants’ perceptions of artificial intelligence accuracy, time efficiency, training needs, and clinical adoption.

Multimedia Appendix 8 Course structure and content.

## Abbreviations

AI: artificial intelligence
GP: general practitioner
LLM: large language model
MCQ: multiple-choice question

## Data Availability

All data generated or analyzed during this study are included in this published paper and its multimedia appendices.
