# Supplementary material for: Effectiveness of Informed AI Use on Clinical Competence of General Practitioners and Internists: Pre-Post Intervention Study
Source: JMIR Med Educ. 2026 Feb 5;12:e75534. doi: 10.2196/75534 (PMC12921430; doi:10.2196/75534)
Supplement: Multimedia Appendix 1 [file mededu_v12i1e75534_app1.docx]

***Multimedia Appendix 1***

***Classification of Prior AI Us:****:*

- To compare test performance based on prior AI use in clinical practice, participants who reported never, or rarely using AI were classified as ‘unfamiliar’ with AI. Those who reported using AI sometimes, frequently or almost every day were classified as ‘familiar.
