# Supplementary material for: Effectiveness of Informed AI Use on Clinical Competence of General Practitioners and Internists: Pre-Post Intervention Study
Source: JMIR Med Educ. 2026 Feb 5;12:e75534. doi: 10.2196/75534 (PMC12921430; doi:10.2196/75534)
Supplement: Multimedia Appendix 2 [file mededu_v12i1e75534_app2.docx]

***Multimedia Appendix 2***

***Recruitment:***

- Participants were recruited through professional networks and social media platforms, interested individuals voluntarily completed a registration form, which required upload of medical licensure to confirm eligibility. Those deemed eligible were enrolled into the study. Recruitment was conducted over a three-month period, ending in March 2025. During this period, study enrollment, course access, and both pre- and post-tests and perception assessments were available to participants.
