# Supplementary material for: Effectiveness of Informed AI Use on Clinical Competence of General Practitioners and Internists: Pre-Post Intervention Study
Source: JMIR Med Educ. 2026 Feb 5;12:e75534. doi: 10.2196/75534 (PMC12921430; doi:10.2196/75534)
Supplement: Multimedia Appendix 3 [file mededu_v12i1e75534_app3.docx]

*Multimedia Appendix 3*

Table of Contents

[Sample Case 1: Chronic Cough 2](#_Toc214131834)

[1. Diagnosis & Patient Assessment (S1) 2](#_Toc214131835)

[2. Diagnosis & Patient Assessment (S1) 3](#_Toc214131836)

[3. Treatment Planning & Personalized Medicine (S2) 3](#_Toc214131837)

[Sample Case 2: Acute Knee Swelling 4](#_Toc214131838)

[1. Treatment Planning & Personalized Medicine (S2) 5](#_Toc214131839)

[2. Diagnosis & Patient Assessment (S1) 5](#_Toc214131840)

[3. Treatment Planning & Personalized Medicine (S2) 6](#_Toc214131841)

[Sample Case 3: Polycythemia With Thrombosis Risk 7](#_Toc214131842)

[1. Diagnosis & Patient Assessment (S1) 7](#_Toc214131843)

[2. Diagnosis & Patient Assessment (S1) 8](#_Toc214131844)

[Sample Case 4: Screening Discovery of Type 2 Diabetes 9](#_Toc214131845)

[1. Treatment Planning & Personalized Medicine (S2) 9](#_Toc214131846)

[2. Diagnosis & Patient Assessment (S1) 10](#_Toc214131847)

[3. Diagnosis & Patient Assessment (S1) 10](#_Toc214131848)

[Sample Case 5: Fever in a Chemotherapy Patient 11](#_Toc214131849)

[1. Treatment Planning and Personalized Medicine (S2) 12](#_Toc214131850)

[2. Diagnosis and Patient Assessment (S1) 12](#_Toc214131851)

[3. Treatment Planning and Personalized Medicine (S2) 13](#_Toc214131852)

[4. Discharge Planning, and Patient Counseling (S3) 13](#_Toc214131853)

[Source References 14](#_Toc214131854)

# Sample Case 1: Chronic Cough

Clinical Scenario

A 19-year-old male reports a long history of persistent cough and recurrent chest infections since early childhood. Over the past two years, symptoms have intensified, leading to repeated hospital admissions for productive cough and purulent sputum. Past sputum cultures have grown Haemophilus influenzae twice, and his most recent hospitalization revealed Pseudomonas aeruginosa. Despite partial response to antibiotics, he continues to produce daily sputum. He has no family history of lung disease.**On exam,** he is thin, with a few fine inspiratory crackles over both upper lung zones. Cardiovascular and abdominal exams are normal.

MCQs

### Diagnosis & Patient Assessment (S1)

Which genetic abnormality is most commonly associated with this patient’s likely underlying disease?

A. CFTR ΔF508 mutation

B. BCR–ABL translocation

C. ALK gene rearrangement

D. EGFR mutation

E. JAK2 V617F mutation

Correct answer: A

### 2. Diagnosis & Patient Assessment (S1)

What imaging modality best characterizes the extent of structural lung damage?

A. Chest X-ray

B. High-resolution CT

C. MRI

D. PET-CT

E. Lung ultrasound

Correct answer: B

### 3. Treatment Planning & Personalized Medicine (S2)

What is the most appropriate initial disease-modifying therapy?

A. Inhaled corticosteroids

B. Elexacaftor–Tezacaftor–Ivacaftor

C. Bronchoscopy with lavage

D. Lung transplantation

E. Gene replacement therapy

Correct answer: B

# Sample Case 2: Acute Knee Swelling

Clinical Scenario

An 80-year-old woman presents with a two-day history of acute pain, warmth, and swelling in her left knee. She has known mild osteoarthritis of the hips and is on low-dose bendroflumethiazide for hypertension. On exam, the affected knee is warm, visibly swollen, and tender, with limited flexion. Her right knee is normal.

Investigations

- Haemoglobin: 12.1 g/dL (normal: 11.7–15.7 g/dL)
- White cell count: 12.4 × 10⁹/L (normal: 3.5–11.0 × 10⁹/L)
- Platelets: 384 × 10⁹/L (normal: 150–440 × 10⁹/L)
- Erythrocyte sedimentation rate (ESR): 48 mm/h (normal: <10 mm/h)
- Sodium: 136 mEq/L (normal: 135–145 mEq/L)
- Potassium: 3.6 mEq/L (normal: 3.5–5.0 mEq/L)
- Urea: 20.28 mg/dL (normal: 5-20 mg/dL)
- Creatinine: 1.3 mg/dL (normal: 0.6–1.1 mg/dL)
- Glucose: 194 mg/dL (normal: 70–100 mg/dL)

MCQs

### 1. Treatment Planning & Personalized Medicine (S2)

What is the most appropriate approach to manage her acute joint pain?

A. Start NSAIDs without precautions

B. Start NSAIDs and add a PPI

C. Use corticosteroids alone

D. Begin physical therapy

E. Prescribe opioid analgesics

Correct answer: B

### 2. Diagnosis & Patient Assessment (S1)

Which investigation is required to confirm the diagnosis?

A. Joint aspiration with synovial fluid analysis

B. Serum electrolytes

C. ANA testing

D. CRP monitoring alone

E. Serum CK measurement

Correct answer: A

### 3. Treatment Planning & Personalized Medicine (S2)

Given her thiazide use and new joint symptoms, what is the most appropriate antihypertensive adjustment?

A. Continue thiazide and start NSAIDs

B. Switch thiazide to an ARB

C. Increase thiazide dose

D. Stop all antihypertensives

E. Add beta-blocker

Correct answer: B

# Sample Case 3: Polycythemia With Thrombosis Risk

Clinical Scenario

A 55-year-old woman reports progressive headaches, dizziness, and occasional visual blurring over six months. Her vitals are stable, but she appears flushed. Examination reveals a palpable liver edge and asymmetrical swelling of the right leg. Fundoscopy shows engorged retinal veins. Laboratory testing shows elevated hemoglobin and hematocrit with moderately increased platelets.

MCQs

### 1. Diagnosis & Patient Assessment (S1)

Which test confirms the suspected diagnosis?

A. Serum erythropoietin

B. JAK2 mutation assay

C. Peripheral smear

D. Bone marrow biopsy

E. Metabolic panel

Correct answer: B

### 2. Diagnosis & Patient Assessment (S1)

Which factor indicates highest risk for disease progression?

A. Female sex

B. Age 55

C. Presence of right-leg DVT

D. Hemoglobin 17 g/dL

E. Mild leukocytosis

Correct answer: C

# Sample Case 4: Screening Discovery of Type 2 Diabetes

Clinical Scenario

A 52-year-old woman presents for her annual physical examination with no active complaints. Her medical history includes borderline hypertension and moderate obesity. One year earlier, her fasting lipid profile was within acceptable limits for someone without major coronary artery disease risk factors. Her family history is significant for diabetes and hypertension in her mother and older brother. She previously received counseling to follow a low-calorie, low-fat diet and increase physical activity, but she has not adopted these changes. She explains that her full-time job and responsibilities for three children limit her ability to exercise, and he}|r family frequently eats meals outside the home.

On examination, her blood pressure is 140/92 mm Hg, and her body mass index is 29 kg/m². Acanthosis nigricans is noted on the posterior neck, while the remainder of the exam is normal. A Pap smear is performed, a mammogram is offered, and because she had not eaten, a fasting plasma glucose test is obtained, measuring 140 mg/dL.

MCQs

### 1. Treatment Planning & Personalized Medicine (S2)

What is the next best step in management?

A. Begin insulin therapy

B. Start metformin and initiate lifestyle changes

C. Repeat fasting glucose only after several weeks

D. Start a sulfonylurea

E. Initiate GLP-1 agonist therapy immediately

Correct answer: B

### 2. Diagnosis & Patient Assessment (S1)

Which test should be ordered next to confirm the diagnosis?

A. Hemoglobin A1C

B. Oral glucose tolerance test

C. Serum insulin level

D. Random plasma glucose

E. C-peptide

Correct answer: A

### 3. Diagnosis & Patient Assessment (S1)

Which factor confers the highest cardiovascular risk?

A. Family history of diabetes

B. BMI 29 kg/m²

C. Diagnosis of type 2 diabetes

D. Sedentary lifestyle

E. BP 140/92 mmHg

Correct answer: C

# Sample Case 5: Fever in a Chemotherapy Patient

Clinical Scenario

A 25-year-old man undergoing chemotherapy for acute lymphoblastic leukemia presents after 12 hours of fever and chills. His latest chemotherapy cycle (cyclophosphamide, vincristine, doxorubicin, and dexamethasone) was administered one week ago. He denies cough, gastrointestinal issues, neurologic symptoms, recent travel, or exposure to ill contacts. He uses a tunneled central venous catheter in the right internal jugular vein and reports flushing it daily. He recalls experiencing a 20–30 minute episode of rigors the previous day shortly after flushing the catheter.

Physical Examination

On arrival, he appears unwell. His temperature is 39.4°C, heart rate 120/min, blood pressure 118/65 mm Hg, and respiratory rate 22/min. His skin is warm and sweaty with no rash. Lung auscultation is clear bilaterally. Cardiac examination reveals a soft systolic murmur at the left sternal border. Abdominal examination is benign. The catheter site shows no redness, tenderness, or discharge.

Laboratory Findings

WBC count is 1100/mm³ with 10% neutrophils, 16% bands, 70% lymphocytes, and 4% monocytes, giving an absolute neutrophil count of 286/mm³.

Questions

### 1. Treatment Planning and Personalized Medicine (S2)

What is the most critical next step in management?

A. Obtain blood cultures from catheter and peripheral vein, then start broad-spectrum antibiotics

B. Remove the central venous catheter immediately

C. Start G-CSF therapy

D. Wait for complete culture results before starting antibiotics

E. Perform CT chest to rule out pneumonia

Correct Answer: A. Obtain blood cultures from catheter and peripheral vein, then start broad-spectrum antibiotics

### 2. Diagnosis and Patient Assessment (S1)

Which of the following factors increases the risk of poor outcomes in this patient with neutropenic fever?

A. White blood cell count of 1100/mm³

B. Recent chemotherapy with hyperfractionated cyclophosphamide

C. Peripheral catheter placement

D. Patient age under 25 years

E. Normal chest radiograph

Answer: B. Recent chemotherapy with hyperfractionated cyclophosphamide

### 3. Treatment Planning and Personalized Medicine (S2)

Which of the following would be most appropriate for future prevention in this patient ?

A. Daily prophylactic catheter flushing with antibiotics

B. Monthly catheter replacement

C. Pneumococcal and influenza vaccination

D. Daily oral antibiotics indefinitely

E. Routine antifungal prophylaxis

Correct Answer: C Pneumococcal and influenza vaccination

### 4. Discharge Planning, and Patient Counseling (S3)

Which of the following lifestyle modifications should be recommended to this patient upon discharge to prevent future infections?

A. Avoid crowded places and sick contacts

B. Stop flushing the catheter daily

C. Discontinue chemotherapy temporarily

D. Reduce fluid intake to avoid catheter contamination

E. Increase protein intake to boost immunity

Answer: A. Avoid crowded places and sick contacts

# Source References

1. American College of Rheumatology Guidelines for the Management of Systemic Lupus Erythematosus, 2021.
2. BMJ. (2012). Gout management. BMJ, 344, d8190. https://doi.org/10.1136/bmj.d8190
3. EULAR (European League Against Rheumatism) recommendations for the management of systemic lupus erythematosus: 2023 update.
4. Juraschek SP, Simpson LM, Davis BR, et al. The effects of antihypertensive class on gout in older adults: J Hypertens. 2020 May;38(5):954-960.
5. Rees, P. J., Pattison, J., & Kosky, C. (2017). *100 cases in clinical medicine* (3rd ed.). CRC Press.
6. Shteinberg, M., & Taylor‐Cousar, J. L. (2023). The Promise and Pitfalls of CFTR Modulators for Cystic Fibrosis. *Pharmaceuticals*, 16(3), 410. https://doi.org/10.3390/ph16030410
7. Toy, E. C., & Aisenberg, G. M. (2021). *Case files: Internal medicine* (6th ed.). McGraw Hill.
8. Willner, D. A., Goyal, A., Grigorova, Y., & Treasure, C. (2024). Pericardial effusion. In *StatPearls* [Internet]. StatPearls Publishing. https://www.ncbi.nlm.nih.gov/books/NBK431090/
