# Supplementary material for: Effectiveness of Informed AI Use on Clinical Competence of General Practitioners and Internists: Pre-Post Intervention Study
Source: JMIR Med Educ. 2026 Feb 5;12:e75534. doi: 10.2196/75534 (PMC12921430; doi:10.2196/75534)
Supplement: Multimedia Appendix 4 [file mededu_v12i1e75534_app4.docx]

***Multimedia Appendix 4***

**Supplemental Table 1:** Evaluation of crossover design (n= 326)

| **Evaluation of crossover design (n= 326)** | | | | | |
| --- | --- | --- | --- | --- | --- |
| **Parameter** | **Exam A**  **median (IQR)** | **Exam B**  **median (IQR)** |  | | **p-value** |
|  | | | **U** | **Z** |  |
| Score before (%) | 56.00 (44.00-68.00) | 57.00 (47.85-69.60) | 12889.00 | -.456 | .648^a^ |
| Score After (%) | 80.00 (72.00-88.00) | 82.65 (73.95-87.00) | 12865.00 | -.449 | .653^a^ |

|  | **Mean (SD)** | |  |  |  |
| --- | --- | --- | --- | --- | --- |
|  | A→B | B→A | **Difference between the differences** | **t(df)** |  |
| Difference between average scores | 22.16 (18.43) | 19.13 (19.68) | 3.03 | 1.44 (324) | .152^b^ |

| **Pre-Test** | | | | |
| --- | --- | --- | --- | --- |
|  | **Exam A** | **Exam B** | **Total** |  |
| Fail | 154 (92.2%) | 151 (95.0%) | 305 (93.6%) | 0.312^c^ |
| Pass | 13 (7.8%) | 8 (5.0%) | 21 (6.4%) |  |
|  | 167 | 159 | 326 |  |

| **Post-Test** | | | | |
| --- | --- | --- | --- | --- |
|  | **Exam A** | **Exam B** | **Total** |  |
| Fail | 60 (37.7%) | 75 (44.9%) | 135 (41.4%) | 0.189^c^ |
| Pass | 99 (62.3%) | 92 (55.1%) | 191 (58.6%) |  |
|  | 159 | 167 | 326 |  |

^a^ Mann-Whitney Test; ^b^ Independent Samples t-Test; ^c^Chi-Square Test

**Supplemental Table 2:** Who benefited more from informed AI use (n= 326)

| **Who benefited more from the course (n= 326)** | | | | | | | | |
| --- | --- | --- | --- | --- | --- | --- | --- | --- |
| **Parameter** | |  |  | | | | | **p-value** |
|  | | **Score differences mean (SD)** | **Mean difference** | | | **t(df)** | |  |
| Gender | Male (n, %) | 19.62 (18.0) | -2.62 | | | -1.22 (324) | | 0.223^a^ |
|  | Female (n, %) | 22.24 (20.39) |  |  |  |  |  |  |
| Specialty | GP | 23.65 (19.07) | 9.95 | | | 4.27 (324) | | <0.001***^a^ |
|  | Internist | 13.69 (17.26) |  |  |  |  |  |  |
| Received previous training in AI | Yes | 22.23 (17.27) | 1.63 | | | .35 (324) | | .725^a^ |
|  | No | 20.60 (19.20) |  |  |  |  |  |  |
| Familiarity with AI | Not familiar | 20.98 (20.07) | .81 | | | .37 (324) | | .713^a^ |
|  | Familiar | 20.17 (17.27) |  |  |  |  |  |  |
|  |  |  | **df** | **ss** | **MS** | | **F** |  |
| Type of served patients | Outpatients | 22.47 (20.85) | **2** | **442.61** | **221.31** | | **.607** | **.546^b^** |
|  | Inpatients | 19.59 (20.82) |  |  |  |  |  |  |
|  | Both | 20.69 (19.08) |  |  |  |  |  |  |

^a^ Independent Samples t-Test; ^b^ ANOVA (Analysis of Variance)

***p<0.001 is statistically extremely significant

**Supplemental Table 3:** Time per correct answer per skill in seconds (n= 326)

| **Time per correct answer per skill in seconds (n= 326)** | | | | | |
| --- | --- | --- | --- | --- | --- |
| **Parameter** | **Time per correct answer before (sec)**  **median (IQR)** | **Time per correct answer after (sec)**  **median (IQR)** | **z** | **p-value** | **r** |
| Skill I (diagnosis) | 45.00 (27.00 - 77.25) | 67.00 (49.75 - 93.25) | -6.24 | <0.001^***a^ | 0.346 |
| Skill II (treatment) | 50.50 (27.75 - 84.25) | 75.00 (50.75 - 102.00) | -4.89 | <0.001^***a^ | 0.273 |
| Skill III (counselling) | 34.50 (18.00 - 47.00) | 47.00 (29.00 - 68.25) | -6.21 | <0.001^***a^ | 0.345 |

^a^ Wilcoxon Signed Ranks Test; r: effect size

***p<0.001 is statistically extremely significant

**Supplemental Table 4:** Responses to perception questions (n= 250)

| **Perception responses (n= 250)** | | | | |
| --- | --- | --- | --- | --- |
| **Question** | **Responses** | **Code** | **Before (n,%)** | **After (n,%)** |
| Assuming optimal use of the best available Artificial Intelligence platform, how accurate do you think it would be in diagnosis, risk calculation, and recommendation of additional tests? | More than 80% of the time | 3 | 61 (24.4%) | 166 (66.4%) |
|  | 50–80% of the time | 2 | 131 (52.4%) | 81 (32.4%) |
|  | Less than 50% of the time | 1 | 35 (14.0%) | 3 (1.2%) |
|  | I don’t know | 0 | 23 (9.2%) | - |
| Assuming optimal use of the best available Artificial Intelligence platform, how accurate do you think it would be in treatment planning and personalized medicine? | More than 80% of the time | 3 | 44 (17.6%) | 159 (63.6%) |
|  | 50–80% of the time | 2 | 129 (51.6%) | 84 (33.6%) |
|  | Less than 50% of the time | 1 | 58 (23.2%) | 7 (2.8%) |
|  | I don’t know | 0 | 19 (7.6%) | - |
| In your opinion, how much time can AI technologies save healthcare professionals in clinical settings? | More than 50% | 2 | 144 (57.6%) | 205 (82.0%) |
|  | Less than 50% | 1 | 71 (28.4%) | 39 (15.6%) |
|  | No time saving/ I don’t know | 0 | 35 (14.0%) | 6 (2.4%) |
| To what extent do you think structured training program can enhance physicians' effectiveness in utilizing AI? | Significantly | 3 | 167 (66.8%) | 205 (82.0%) |
|  | Moderately | 2 | 64 (25.6%) | 43 (17.2%) |
|  | Slightly | 1 | 7 (2.8%) | 2 (0.8%) |
|  | No enhancement/ Not sure | 0 | 12 (4.8%) | - |
| How willing and confident do you feel about incorporating AI tools into your clinical practice at this time?" | Very willing and confident | 3 | 86 (34.4%) | 136 (54.4%) |
|  | Somewhat willing and confident | 2 | 109 (43.6%) | 103 (41.2%) |
|  | Slightly willing and confident | 1 | 48 (19.2%) | 11 (4.4%) |
|  | Not at all willing or confident | 0 | 7 (2.8%) | - |

**Supplemental Table 5:** Comparison of the participants’ perception before and after informed use of AI (n= 250)

| **Comparison of the participants’ perception before and after the course (n= 250)** | | | | |
| --- | --- | --- | --- | --- |
| **Question** | **Perception before**  **median (IQR)** | **Perception after**  **median (IQR)** | **z** | **p-value** |
| Assuming optimal use of the best available Artificial Intelligence platform, how accurate do you think it would be in diagnosis, risk calculation, and recommendation of additional tests? | 2 (2 - 2) | 3 (2 – 3) | -10.20 | <0.001^***a^ |
| Assuming optimal use of the best available Artificial Intelligence platform, how accurate do you think it would be in treatment planning and personalized medicine? | 2 (1 – 2) | 3 (2 – 3) | -10.88 | <0.001^***a^ |
| In your opinion, how much time can AI technologies save healthcare professionals in clinical settings? | 2 (1 – 2) | 2 (2 – 2) | -6.94 | <0.001^***a^ |
| To what extent do you think structured training program can enhance physicians' effectiveness in utilizing AI? | 3 (2 – 3) | 3 (3 – 3) | -5.00 | <0.001^***a^ |
| How willing and confident do you feel about incorporating AI tools into your clinical practice at this time?" | 2 (2 – 3) | 3 (2 – 3) | -7.27 | <0.001^***a^ |

^a^ Wilcoxon Signed Ranks Test

***p<0.001 is statistically extremely significant

**Supplemental Table 6:** Perception difference between the groups (n= 250)

| **Perception difference between the groups (n = 250) (p value)** | | | | | | |
| --- | --- | --- | --- | --- | --- | --- |
| **Parameter** | | **Assuming optimal use of the best available Artificial Intelligence platform, how accurate do you think it would be in diagnosis, risk calculation, and recommendation of additional tests?** | **Assuming optimal use of the best available Artificial Intelligence platform, how accurate do you think it would be in treatment planning and personalized medicine?** | **In your opinion, how much time can AI technologies save healthcare professionals in clinical settings?** | **To what extent do you think structured training program can enhance physicians' effectiveness in utilizing AI?** | **How willing and confident do you feel about incorporating AI tools into your clinical practice at this time?"** |
| Gender^a^ | Male | .356 | .580 | .175 | .302 | .909 |
|  | Female |  |  |  |  |  |
| Specialty^a^ | GP | .294 | .796 | .433 | .913 | .907 |
|  | Internist |  |  |  |  |  |
| Familiarity with AI^a^ | Non-familiar | .011*^c^ | .051 | .109 | .061 | <0.001***^c^ |
|  | Familiar |  |  |  |  |  |
| Received previous training in AI^a^ | Yes | .257 | .815 | .599 | .740 | .808 |
|  | No |  |  |  |  |  |
| Type of served patients^b^ | Outpatients | .602 | .688 | .258 | .307 | .840 |
|  | Inpatients |  |  |  |  |  |
|  | Both |  |  |  |  |  |

^a^ Mann-Whitney Test; ^b^ Kruskal Wallis Test

^c^  Favor toward non-familiar (non-familiar benefited more)

* *p*<0.05 is statistically significant; **p<0.01 is statistically very significant; ***p<0.001 is statistically extremely significant
