# Supplementary material for: Effectiveness of Informed AI Use on Clinical Competence of General Practitioners and Internists: Pre-Post Intervention Study
Source: JMIR Med Educ. 2026 Feb 5;12:e75534. doi: 10.2196/75534 (PMC12921430; doi:10.2196/75534)
Supplement: Multimedia Appendix 5 [file mededu_v12i1e75534_app5.docx]

***Multimedia Appendix 5***

***Passing Score Rationale:***

- In alignment with the high standards adopted by leading medical education and certification bodies, the passing score was set at 80%—a threshold comparable to that of the American **Heart** Association (AHA), which requires 84% for Basic Life Support (BLS) certification ([cpr.heart.org/en/-/media/CPR-Files/2025-documents-for-cpr-heart-edits-posting/Courses-Kits/2025-Guidelines-FAQ-BLS-ILT-%284%29.pdf?sc_lang=en&utm_source=chatgpt.com](https://cpr.heart.org/en/-/media/CPR-Files/2025-documents-for-cpr-heart-edits-posting/Courses-Kits/2025-Guidelines-FAQ-BLS-ILT-%284%29.pdf?sc_lang=en&utm_source=chatgpt.com)), and the American Board of Surgery (ABS), which sets an 80% passing score for Continuous Certification Assessments (CCA) ([Continuous Certification - American Board of Surgery](https://www.absurgery.org/stay-certified/continuous-certification-assessments/?utm_source=chatgpt.com)).
