# Supplementary material for: Effectiveness of Informed AI Use on Clinical Competence of General Practitioners and Internists: Pre-Post Intervention Study
Source: JMIR Med Educ. 2026 Feb 5;12:e75534. doi: 10.2196/75534 (PMC12921430; doi:10.2196/75534)
Supplement: Multimedia Appendix 6 [file mededu_v12i1e75534_app6.docx]

***Multimedia Appendix 6***

***AI Usage Instructions and Technical Setup:***

- Clear guidance was provided on AI usage in assessments, where participants were required not to use AI during the pre-course test.
- To ensure that participants using free-tier GPT accounts did not exceed their allocated usage and become restricted to limited features during the post-course test, they were instructed to refrain from using their accounts 24 hours before the assessment.
- Additionally, since the free-tier accounts allow only three image uploads per day, the number of medical images included in the tests was limited to three to prevent technical disruptions.
- To minimize variability in completion time due to device-related factors, participants were instructed to use the same type of device (e.g., laptop, tablet, or mobile) for both the pre- and post-course tests.
- The assessment platform, ProProfs®, automatically recorded the time spent per question and total test duration.
- The post-course perception assessment was deliberately conducted after the clinical scenario test so that participants’ views would be informed by their objective performance improvements rather than abstract opinions.
