# Supplementary material for: Effectiveness of Informed AI Use on Clinical Competence of General Practitioners and Internists: Pre-Post Intervention Study
Source: JMIR Med Educ. 2026 Feb 5;12:e75534. doi: 10.2196/75534 (PMC12921430; doi:10.2196/75534)
Supplement: Multimedia Appendix 7 [file mededu_v12i1e75534_app7.docx]

***Multimedia Appendix 7***

1. Assuming optimal use of the best available Artificial Intelligence platform, how accurate do you think it would be in diagnosis, risk calculation, and recommendation of additional tests?

A. More than 80% of the time 3

B. 50–80% of the time 2

C. Less than 50% of the time 1

D. I don’t know 0

2. Assuming optimal use of the best available Artificial Intelligence platform, how accurate do you think it would be in treatment planning and personalized medicine?

A. More than 80% of the time 3

B. 50–80% of the time 2

C. Less than 50% of the time 1

D. I don’t know 0

3. In your opinion, how much time can AI technologies save healthcare professionals in clinical settings?

A. More than 50% 2

B. Less than 50% 1

E. No time saving 0

D. I don’t know 0

4. To what extent do you think structured training program can enhance physicians' effectiveness in utilizing AI?

1. Significantly 3
2. Moderately 2
3. Slightly 1
4. No enhancement 0
5. Not sure 0

5. How willing and confident do you feel about incorporating AI tools into your clinical practice at this time?"

A. Very willing and confident 3

B. Somewhat willing and confident 2

C. Slightly willing and confident 1

D. Not at all willing or confident 0
