# Supplementary material for: Effectiveness of Informed AI Use on Clinical Competence of General Practitioners and Internists: Pre-Post Intervention Study
Source: JMIR Med Educ. 2026 Feb 5;12:e75534. doi: 10.2196/75534 (PMC12921430; doi:10.2196/75534)
Supplement: Multimedia Appendix 8 [file mededu_v12i1e75534_app8.docx]

***Multimedia Appendix 8***

**Course Title: AI Skills in Medicine**

**Module 1: Overview of AI Medical Applications**

**Learning Objectives:**

By the end of this module, participants were expected to:

- Understand, through practical demonstrations, how AI enhances diagnostic accuracy, risk prediction, treatment planning, and patient management across key clinical domains.
- Apply structured prompting to obtain clinically meaningful and guideline-aligned outputs from AI tools.
- Appreciate the benefits and limitations of AI assistance in real-world clinical reasoning, particularly in resource-limited settings.

**Module Description:**

This module provided a **broad but practical overview** of AI medical applications, constituting the **main instructional component** of the 1.5-hour course. Through concise demonstrations and brief case discussions, participants were introduced to how AI can augment, but not replace, clinical reasoning across key medical functions.

**Module Content (Conceptual Overview with Illustrative Cases):**

- 1. **AI in Diagnosis and Patient Assessment**

This section provided a concise overview of diagnostic applications, primarily to familiarize participants with the scope of AI use in clinical reasoning.

- *Clinical Decision Support:* Briefly introduced how AI can generate differential diagnoses from patient data.
- *Diagnostic Test Selection:* Mentioned AI’s potential role in recommending appropriate confirmatory or exclusionary investigations.
- *Medical Imaging and Laboratory Interpretation:* Touched on AI’s capacity to detect subtle imaging or laboratory abnormalities.
  1. **Predictive Analytics for Patient Outcomes (Case-Based Demonstration)**

Two case studies were used to demonstrate structured AI prompting for outcome prediction and risk analysis:

**Case 1: Thoracic Aortic Aneurysm Progression:**

Participants were shown three anonymized reports (2021–2024) for a 72-year-old woman with a 5.8 cm ascending thoracic aortic aneurysm and tortuous aorta.

They practiced structured prompts to:

- Estimate the **timeframe for potential life-threatening complications** without surgery.
- Assess **surgical risk and complication probability** at current disease status.
- Generate **Outcome-Action Pairings**; linking predicted outcomes to evidence-based recommendations following current guidelines.
- Integrate new anthropometric data (BMI, comorbidities) to update risk assessments and preoperative management advice.
- Identify **modifiable risk factors** to reduce perioperative risk.

**Case 2: Prostate Cancer Relapse Risk:**

Participants prompted AI to:

- Compare two clinical scenarios differing in PSA, Gleason score, and stage.
- Estimate **5-year relapse risk** and recommend appropriate **follow-up tests** and **monitoring frequency.**
- Observe how AI integrates risk stratification and guideline-based follow-up recommendations.

**C. AI in Treatment Planning and Personalized Medicine**

- Conceptual overview of how AI supports evidence-based treatment selection and individualized pharmacotherapy adjustment using patient-specific factors (e.g., pharmacogenomics, renal or hepatic function), with emphasis on balancing benefit–risk ratios (NNT vs NNH).

**D. AI in Clinical Data Extraction**

- Briefly introduced AI’s potential to summarize electronic health records (EHRs) and identify key elements such as drug interactions, missed diagnoses, and dosing errors, without practical demonstration.

**E. AI in Triaging (Applied Example)**

A short exercise demonstrated AI’s utility in prioritizing emergency cases under constrained resources:
Participants were prompted with the following scenario:

*“As an ER doctor with limited resources and one nurse, how would you triage the following cases?”*

- **Patient 1:** 65-year-old male, acute chest pain radiating to left arm, sweating, SOB; history of diabetes and hypertension.
- **Patient 2:** 45-year-old female, fever 38.5°C, dry cough, mild SOB for 3 days.
- **Patient 3:** 25-year-old male, right ankle injury while playing basketball, partial weight-bearing.

This exercise highlighted how AI can rapidly prioritize **critical vs non-urgent** cases and assist in admission and management decisions.

**Module 2: Optimal Use of AI for Clinical Practice**

**Learning Objectives:**By the end of this module, participants were expected to:

- Identify when AI use may or may not be needed in clinical cases.
- Use AI tools thoughtfully to support, not replace, clinical reasoning.
- Check the accuracy and reliability of AI-generated answers and references.

**Module Description:**

This module focused on the **practical principles of responsible AI utilization** in everyday clinical contexts. It emphasized clinical judgment as the starting point for any case analysis, with AI positioned as an *adjunctive, consultative tool* rather than a replacement for professional reasoning. The content was presented through brief illustrated guidance followed by a short applied exercise.

**Module Content:**

1. **Differentiate Case Complexity:** Identify straightforward cases that do not require AI support versus complex cases where AI can add value.
2. **Begin with Clinical Reasoning:** Formulate an initial diagnostic or management plan using one’s own clinical knowledge before consulting AI.
3. **Engage AI Thoughtfully:** Use AI (e.g., ChatGPT-4) for a second opinion or to expand differential diagnoses.
4. **Analyze AI responses:** Evaluate the rationale behind the AI’s suggestions.
5. **Request Clarification:** If needed, ask AI for evidence or references supporting its claims.
6. **Validate Sources:** Verify that cited references are genuine, recent, and evidence-based.
7. **Maintain Clinical Primacy:** Treat AI as a *decision-support* aid, not a substitute for guidelines or professional judgment.

**Applied Exercise: Recurrent Peptic Ulcer Symptoms Post-Treatment:**
Participants were given the following prompt to apply the above principles:

“A 45-year-old Jordanian male presents with recurrent epigastric pain, bloating, and nausea. He was treated for *Helicobacter pylori* with triple therapy (omeprazole 20 mg BID, clarithromycin 500 mg BID, and amoxicillin 1 g BID for 14 days), achieving temporary symptom resolution. Symptoms recurred, and he was prescribed levofloxacin-based therapy (omeprazole 20 mg BID, levofloxacin 500 mg QD, and amoxicillin 1 g BID for 10 days), but relapse occurred six weeks later.”

Participants were guided to:

- Formulate an initial assessment independently.
- Prompt AI for possible causes of treatment failure and management options.
- Evaluate AI’s responses for diagnostic accuracy, alignment with updated *H. pylori* management guidelines, and validity of cited references.

The exercise reinforced **selective AI use, critical validation of outputs, and maintenance of clinician oversight** in therapeutic decision-making.

**Module 3: Privacy and Ethical Considerations**

**Learning Objectives:**
By the end of this brief module, participants were expected to:

- Understand the main risks of data exposure and shared account use when employing AI tools.
- Apply simple best practices to protect patient confidentiality and account security.
- Recognize personal accountability for clinical decisions made with AI assistance.

**Module Description:**
This was a concise module introducing key privacy and ethical considerations in AI-assisted clinical practice. It aimed to raise participants’ awareness of essential principles of data protection, account security, and professional responsibility, emphasizing the safe and accountable use of AI tools in healthcare settings.

**Module Content (Overview):**

1. **1. Can Data Simply Leak?** Clarified that information entered by one user cannot be directly accessed by others, addressing common misconceptions about cross-user data exposure.
2. **Account Security:** Emphasized that sharing login credentials can compromise patient confidentiality and expose sensitive information.
3. **Institutional Account Risks:** Explained the risk of using shared hospital accounts, particularly when staff members change or leave the workplace.
4. **Best Practices:** Highlighted key preventive steps: avoid entering identifiable patient data, update shared passwords, and enforce secure access protocols.
5. **Accountability:** Reinforced that clinicians remain fully responsible for clinical decisions made with AI assistance, regardless of tool accuracy.

**Module 4: Customizing Your AI Account**

**Learning Objective:**
Understand that account customization can improve the relevance and professionalism of AI responses.

**Module Description:**
This short closing module briefly showed how setting user identity and response preferences in ChatGPT enhances output quality.

**Key Points:**

- Specify professional role (e.g., “I am a cardiologist”) to tailor responses.
- Define tone and format (concise, structured, evidence-based).
- Set preferences for scientific integrity, depth, and visual clarity.
